# Supplementary material for: Hemispherotomy in Infants with Hemimegalencephaly: Long-Term Seizure and Developmental Outcome in Early Treated Patients
Source: Brain Sci. 2022 Dec 30;13(1):73. doi: 10.3390/brainsci13010073 (PMC9856354; doi:10.3390/brainsci13010073)
Supplement: Supplementary file 1 [file brainsci-13-00073-s001.zip › brainsci-2087481-supplementary.pdf]

**GMDS**	DQ	DA
General	62	10 months
Locomotor Area	78	12,5 months
Personal and Social Area	65	10,5 months
Language Area	62	10 months
Manual and Visual Coordination Area	59	9,5 months
Non-Verbal Skill Area	46	7,5 months

Supplementary Table S1. **Developmental presurgical scale of patient #1 at 16 months**.

GMDS: Griffiths Mental Developmental Scales, DQ: Developmental Quotient, DA: Developmental Age

**WISC IV**	Index
Full scale IQ	**73**
Verbal Comprehension Index	92
Perceptual Reasoning Index	74
Working Memory Index	67
Processing Speed Index	82

Supplementary Table S2: Cognitive postsurgical evaluation of patient #1 at 10,5 years (9 years after surgery)

GMDS	Pre-surgery		Post-surgery	
	**DQ (30 m)**	**DA (30 m)**	**DQ (61m)**	**DA (61m)**
General	**53**	16 m	**53**	32 m
Locomotor Area	53	16 m	37	22,5 m
Personal and Social Area	60	18 m	64	39 m
Language Area	43	13 m	59	36 m
Manual and Visual Coordination Area	57	17 m	43	26 m
Non-Verbal Skill Area	51	15,5 m	61	37 m

Supplementary Table S3: Pre- and postsurgical GMDS of patient #2

**GMDS**				
Chronological Age	3,5 m	15 m	27 m	48 m
	DQ	DQ	DQ	DQ
Locomotor Area	60	44	26	17
Personal and Social Area	94	51	34	31
Language Area	31	53	35	17
Manual and Visual Coordination Area	n.a.	43	26	21
Non-Verbal Skill Area	57	36	24	35

Supplementary Table S4: Post-surgical GMDS of patient #4 at 3.5, 15, 27 and 48 months after surgery
